# Prolactin-activated PAK1 potentiates estrogen response to breast cancer cell epithelial-mesenchymal transition, migration and invasion.

**DOI:** 10.17912/micropub.biology.001195

**Published:** 2024-06-11

**Authors:** Alan Hammer, Maria Diakonova

**Affiliations:** 1 Department of Biological Sciences, University of Toledo, Toledo, Ohio, United States

## Abstract

Hormones estrogen and prolactin exert independent effects on breast cancer while their crosstalk synergistically enhance breast cancer cell proliferation. We have previously shown that the serine/threonine kinase PAK1 is responsible for this effect and proposed the mechanism of PAK1 action. Here we extended our previous data to demonstrate that the PAK1 kinase is a common interplay in PRL and E2 crosstalk to regulate epithelial-mesenchymal transition, cell migration and invasiveness of human breast cancer cells.

**Figure 1. Prolactin-activated PAK1 potentiates estrogen response to breast cancer cell epithelial-mesenchymal transition, migration and invasion:**
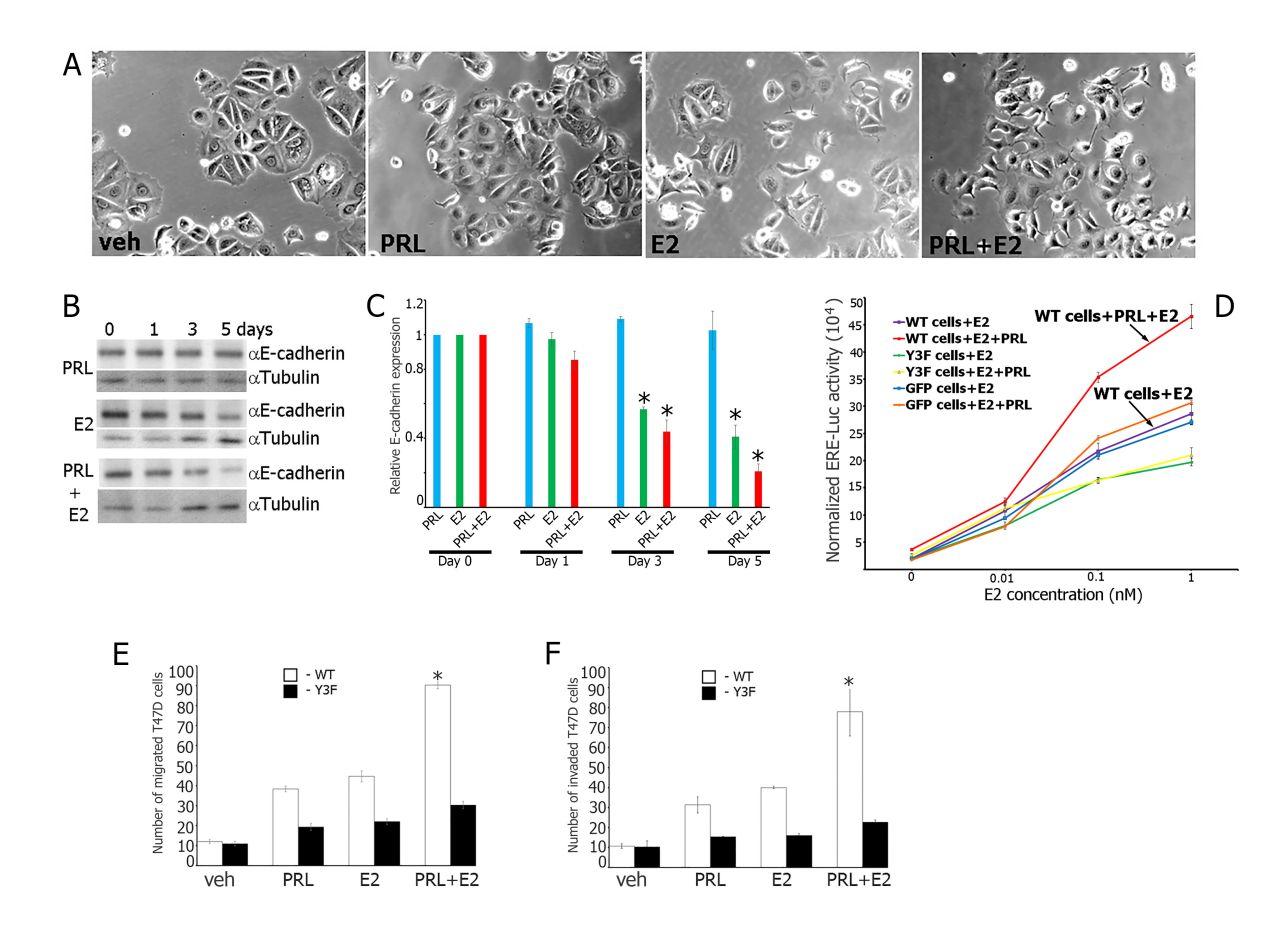
**A)** T47D cells acquired mesenchymal-like phenotype in response to PRL+ E2 in 5 days. T47D cells were treated with either vehicle (veh), prolactin (PRL), estrogen (E2) or prolactin with estrogen (PRL+E2) for 5 days. **B)** Prolactin and estrogen (PRL+E2) down-regulate E-cadherin expression to a higher extent than treatment with E2 only. Parental T47D cells were treated either with PRL (200 ng/ml), E2 (1 nM) or PRL+E2 for 0-5 days. Whole cell lysates were immunoblotted with the indicated antibodies. Anti-gamma-Tubulin antibody was used as a loading control. **C** ) Plot represents the densitometric analysis of the bands obtained for E-cadherin expression at different days of treatment and normalized to E-cadherin expression at day 0. n = 3 for each experimental condition. Bars represent mean ± SE. *P < 0.05 compared with the same treatment at day 0. **D)** PRL-activated tyrosyl-phosphorylated PAK1 potentiates E2 response. T47D clones expressing GFP, PAK1 WT or Y3F were co-transfected with ERE-driven luciferase and β-gal genes (as an internal control). The cells were treated with E2 (0-1 nM) with or without PRL. PAK1 WT clones exhibited the maximal ERE activity in response to E2+PRL. **E-F)** Maximal migration and invasion of T47D in response to PRL+E2 requires tyrosyl phosphorylation of PAK1. T47D cells stably overexpressing PAK1 WT (white bars) or PAK1 Y3F mutant (black bars) were serum deprived, and equal amounts of cells were loaded into the upper part of Boyden chamber uncoated ( **E** ) or coated with the Matrigel ( **F** ). The number of cells that migrates ( **E** ) or invades ( **F** ) to lower surface of chamber toward either veh, PRL, E2 or PRL+E2 after 48 h was counted. Bars represent mean +/- SE. *P<0.05 compared with the same cells treated with either veh, PRL or E2.

## Description

**Description**

Breast cancer is one of the leading causes of deaths in women around the world with tumormetastasis as the main cause. Current therapies have improved breast cancer prognosis, but they often resultin severe side effects. Early detection and better treatment of breast cancer has resulted in remarkable increases in 5-year survival rates; however, ~40,000 women still die from this disease in the U.S. yearly, indicating that improved clinical interventions are needed. Two hormones, prolactin (PRL) and estrogen (E2 for 17-β-estradiol), are absolutely required for normal mammary development and function, while their aberrant signaling is involved in breast cancer. Estrogens play a crucial role in breast cancers, with about 2/3 of all breast tumors expressing estrogen receptor α (ER α; Barone, Brusco et al. 2010). The role of PRL in breast cancer has been confirmed at the cellular level *in vitro* , with multiple transgenic and knockout models *in vivo* , and through epidemiologic analysis (reviewed in Tworoger and Hankinson 2006, Tworoger and Hankinson 2008, Clevenger and Rui 2022, Schuler and O'Leary 2022). In a physiological context, breast cancer cells are exposed to both hormones. However, surprisingly little is known about how PRL and E2 may interact in mammary carcinogenesis, particularly regarding intracellular signaling crosstalk that leads to increased metastasis.

In response to PRL, the prolactin receptor activates the non-receptor tyrosine kinase janus kinase 2 (JAK2), which activates several signal transduction pathways, including p21-activated kinase 1 (PAK1). Members of the serine-threonine kinase PAK family have been implicated in human cancer. Although PAKs are not mutated in cancers, they are overexpressed, hyperactivated or amplified in several human tumors and their role in cell transformation makes them attractive therapeutic targets. Biological studies suggest that PAKs play a key role in proliferative signaling, resisting cell death, activating invasion, metastasis and inducing angiogenesis (reviewed in Rane and Minden 2019, Kumar, Paul et al. 2020, Yao, Li et al. 2020, Rajendran, Swaroop et al. 2022). PAK1 genomic amplification at 11q13 is prevalent in luminal breast cancer and PAK1 protein expression is associated with lymph node metastasis (Ong, Jubb et al. 2011) . Although these observations suggest that PAK1 has a role in breast cancer, its precise role in breast cancer progression remains unknown. PAK1 directly phosphorylates ERα at Serine 305 to promote the transactivation of the receptor leading to upregulation of cyclin D1. This transactivation contributes to the development of E2-independent growth of breast cancer cells and tamoxifen resistance (Rayala and Kumar 2007) . We have previously discovered that PAK1 is a target for PRL-activated JAK2 and that JAK2 phosphorylates PAK1 on Tyrosines 153, 201, and 285 (Rider, Shatrova et al. 2007) . Using phosphospecific antibodies directed to single phosphorylated Tyrosines on PAK1, we identified Tyrosine 285 as a site of PRL-dependent phosphorylation of PAK1 by JAK2 (Hammer, Oladimeji et al. 2015) . We have demonstrated that PRL activates PAK1 in different breast cancer cells (Hammer, Rider et al. 2013, Rider, Oladimeji et al. 2013, Oladimeji, Skerl et al. 2016) . We have also found that, in response to E2, Etk/Bmx kinase phosphorylates Tyrosine 153 on PAK1 and activates PAK1 (Oladimeji, Skerl et al. 2016) . Remarkably, we have shown that PAK1, activated by PRL, phosphorylates Serine 305 on the ER thereby activating ER in the absence of ligand (Oladimeji, Skerl et al. 2016) . This transactivation has been previously shown to contribute to the development of E2-independent growth of breast cancer cells and tamoxifen resistance (Rayala and Kumar 2007) .

Thus, two critical hormone-driven pathways converge on PAK1 via different upstream kinases. In a physiological context, breast cancer cells are exposed to both hormones. Although our previous findings suggest that these hormones crosstalk at the level of PAK1, the extent and consequence of this crosstalk are not known.

Epithelial-mesenchymal transition (EMT) is the initial step for cancer invasion and metastasis. The EMT regulators transform the cancer cells from epithelial-cell like to mesenchymal-cell like with suppression of epithelial markers and expression of mesenchymal markers. The final effects on cancer cells are cancer metastasis, drug resistance and with features of cancer stem cells (Shih and Yang 2011) . One of the first signs that cells have undergone EMT is a change in cell morphology from a polar, well-adherent cell with solid cell-cell interaction to a loosely

adherent, elongated cell with reduced cell-cell interaction. Here we first show that breast cancer epithelial T47D cells demonstrated typical epithelial morphology with developed cell-cell contacts ( **Fig. 1A, veh** ). Treatment of PRL did not cause strong difference in the cell morphology ( **Fig. 1A, PRL** ) while treatment of E2 increased amount of single elongated cells ( **Fig. 1A, E 2** ). Treatment of the cells with both PRL and E2 together (PRL+E2) further reduced cell-cell contacts and promoted elongated cell shape ( **Fig. 1A, PRL +E2** ). This data suggests that PRL+E2 treatment induces to the maximal extent one of the earliest characteristics of EMT: changes of cell shape from an epithelial-like (left image in **Fig. 1A** ) to mesenchymal-like (right image in **Fig. 1A** ).

A hallmark of EMT is the loss of E-cadherin expression (a cell-cell adhesion protein). We treated T47D cells with either PRL (200 ng/ml), E2 (1 nM) or PRL+E2 for 0-5 days and assessed the E-cadherin expression by immunoblotting with anti-E-cadherin antibody ( **Fig. 1B** ). Treatment of PRL did not change amount of E-cadherin in the cell lysates while E2 caused decrease of E-cadherin expression from day 3 ( **Fig. 1C** ). E-cadherin expression was then further diminished in response to PRL+E2 starting at day 3 and exhibiting the minimal expression at day 5. These data demonstrate that PRL+E2 treatment of T47D cells leads to loss of E-cadherin expression to a higher extent than treatment with PRL or E2 separately ( **Fig. 1B** and **C** ).

Several transcriptional factors have been implicated in the transcriptional repression of E-cadherin, including zinc finger proteins, Snail, Slug, Zeb1, Zeb2/Sip1, as well as the basic helix-loop-helix factors Twist and E47 (Batlle, Sancho et al. 2000, Wheelock, Shintani et al. 2008) . PAK1 phosphorylates Snail at Serine 246, resulting in Snail accumulation in the nucleus, enhancement of its repressor function and decreased E-cadherin expression (Yang, Rayala et al. 2005) . Notably, the interaction of PAK1 with Snail in irradiated lung cancer cells was phospho-tyrosyl-dependent and the radiation-induced JAK2-PAK1-Snail signaling pathway increased EMT in these cells (Kim, Youn et al. 2014) . PAK1 has been shown to promote EMT in liver cancer cell lines by increasing Snail expression (Cao and Yin 2020) . Next, we decided to use T47D cell clones stably overexpressing GFP, PAK1 WT or PAK1 Y3F (the clones were characterized previously in Hammer, Rider et al. 2013). PAK1 Y3F mutant is a phospho-tyrosine-deficient PAK1 mutant in which Tyrosines 153, 201, and 285 were mutated to phenylalanines (Rider, Shatrova et al. 2007) . This mutant retains the kinase activity (due to intact kinase domain) but lost the ability to get activated to maximal extent via PRL-dependent tyrosyl phosphorylation (Hammer, Rider et al. 2013) . T47D clones expressing GFP, PAK1 WT or Y3F were co-transfected with ERE-driven luciferase and β-gal genes (as an internal control). The cells were treated with E2 (0-1 nM) with or without PRL. We have shown here that estrogen response element (ERE) -driven reporter gene activity was maximal in PAK1 WT cells treated with E2+PRL, as compared to E2 alone suggesting that PRL-dependent tyrosyl phosphorylation of PAK1 is important for E2-dependent gene expression and that PRL-activated pTyr-PAK1 potentiates E2 response ( **Fig. 1D** ).

We have shown previously that PRL stimulates breast cancer cell migration and invasion via tyrosyl phosphorylation of PAK1 (Hammer, Rider et al. 2013, Rider, Oladimeji et al. 2013, Hammer and Diakonova 2016) . The data in Fig. 1D suggest that pTyr -PAK1 could also play a role in cell motility and invasion in response to PRL+E2. The effect of pTyr-PAK1 on (PRL+E2) - induced cell motility was assessed by evaluating migration through Transwell pores ( **Fig. 1E** ). T47D GFP, PAK1 WT, or PAK1 Y3F cells were serum deprived for 24 h, and equal amounts of cells were loaded into the upper part of the Boyden chamber. The number of cells that migrated to the lower surface of the chamber toward vehicle (veh), PRL, E2 or PRL+E2 were counted in 48 h and plotted. As shown in Fig. 1E, overexpression of PAK 1 WT accelerated migration in response to PRL (>40 cells migrated through the pores) and E2 (> 45 migrated cells) as compared with the cells overexpressing Y3F (around 20 migrated cells for both ligands) whereas migration of PAK1 WT cells was significantly accelerated toward PRL+E2 (> 90 migrated cells) ( **Fig. 1E** ).

Cell migration is a key step in cell invasion so we decided to assess the effect of PRL+E2 on cell invasion. Equal numbers of deprived T47D PAK1 WT and PAK1 Y3F cells were added to the upper part of Boyden chamber coated with Matrigel. Deprivation media with veh, PRL, E2 or PRL+E2 was added to the lower part of the Boyden chamber ( **Fig. 1F** ). The number of cells that invaded through the Matrigel toward ligands was counted. As we have shown previously (Hammer and Diakonova 2016) , PRL stimulated cell invasion to a greater extent in PAK1 WT cells when compared to PAK1 Y3F cells. Invasion toward E2 stimulated cell invasion to the same extent as PRL while (PRL+E2) – induced cell invasion was strongly enhanced in PAK1 WT but not PAK1 Y3F cells.

Thus, our data suggest that the serine-threonine kinase PAK1 is a common node in PRL and E2 crosstalk to synergistically regulate EMT, cell migration and invasiveness of human breast cells.

## Methods

**Cell culture**

Human breast cancer T47D cells were purchased from ATCC. T47D cells stably overexpressing GFP, PAK WT, and PAK1 Y3F were described previously (Hammer, Rider et al. 2013) . The cells were maintained in RPMI 1640 medium (Corning Cellgro, Corning Inc.) supplemented with 10% fetal bovine serum (Sigma Aldrich).

**Assessment of cell morphology** *.* Equal numbers (3 x 10 ^5^ ) of T47D cells were plated into 6-well dishes. Cells were allowed to adhere for 24 h before being washed with PBS and complete media was replaced with deprivation medium (RPMI 1640 medium supplemented with 1% BSA). After 24 h, cells were either treated with veh, PRL (200 ng/ml), E2 (1 nM) or PRL+E2. In 5 days the cells were fixed for 30 min in 4% paraformaldehyde. Phase-contrast images of fixed cells were acquired on an inverted microscope (Olympus IX81) using a PlanApo N 60x/1.42 oil objective lens (Olympus, Tokio, Japan). The experiment was repeated 3 times.

**Western blot analysis** were performed as previously described (Hammer, Rider et al. 2013) . Briefly, T47D cells were deprived of serum for 72 h in deprivation medium (RPMI 1640 medium supplemented with 1% BSA), before treatment with or without PRL (200 ng/ml), and E2 (1 nM) for the indicated times. The cells were rinsed three times with 10 mM sodium phosphate, pH 7.4, 150 mM NaCl, 1 mM Na orthovanadate. Cells were then solubilized in lysis buffer [50 mM Tris (pH 7.5), 0.1% Triton X-100, 150 mM NaCl, 2 mM EGTA, 1 mM Na orthovanadate, 1 mM phenylmethylsulfonyl fluoride, 10 *µ* g/ml aprotinin, 10 *µ* g/ml leupeptin] and centrifuged at 14,000 X *g* for 10 minutes at 4° C. The protein concentration was quantitated using the Bio-Rad protein assay (Bio-Rad Laboratories) and equal amounts of protein were resolved by SDS-PAGE followed by immunoblotting with the indicated antibodies. PVDF patterns were scanned and the amount of E-cadherin was quantified using Multi-Analyst (Bio-Rad Laboratories) software. Relative E-cadherin expression at different days of treatment was than normalized to E-cadherin expression at day 0. Each experimental condition was repeated 3 times.

**Boyden chamber assay.** Cell migration and cell invasion assays were performed as described previously (Hammer, Rider et al. 2013, Rider, Oladimeji et al. 2013, Hammer and Diakonova 2016) . In order to assess the effect of PRL, E2 and PRL+E2 on cell migration, T47D cells stably expressing PAK1 WT, or PAK1 Y3F were serum deprived for 24 h and equal numbers of cells (1 X 10 ^6^ cells/chamber) for each condition were placed in deprivation media in the upper chamber of a Boyden chamber [#07-200-174 (3464), Corning Inc, New York]. Deprivation media with or without PRL (500 ng/ml), E2 (1 nM) or PRL+E2 was placed in the lower chamber. Cells were allowed to migrate for 48 h, after which nonmigrating cells were removed from the upper chamber by a cotton swab. Cells from five separate fields that had migrated through the pores of the membrane to the under- side of the filter were counted after fixation and staining with Different Quik Stain (Polysciences, Inc.). The average amount of cells from five independent fields was plotted. To assess cell invasion, each chamber was coated with collagen IV (Sigma-Aldrich; 1 *µ* g/ml) and processed as described above. Each experimental condition was repeated at least 3 times.

**Luciferase assay**

T47D clones expressing GFP, PAK1 WT or Y3F were co-transfected with ERE-driven luciferase and β-gal genes (as an internal control). The cells were serum deprived and treated with E2 (0-1 nM) with or without PRL (200 ng/ml), lysed, and luciferase activity was measured according to the manufacturer’s protocol (Promega, Madison, WI). Luciferase values were corrected for transient efficiency by determining the ration of luciferase activity to β-galactosidase activity and indicated as “normalized ERE-Luc activity” (Tao, Oladimeji et al. 2011) . Each experimental condition was repeated at least 3 times.

**Statistical analysis**

Data from at least 3 separate experiments per each condition were pooled and analyzed using 1-way ANOVA plus Tukey’s honest significant difference test. Differences were considered to be statistically significant at P < 0.05. Results are expressed as the mean +/- SE.

## Reagents

ERE-Luciferase construct was described earlier (Gutzman, Miller et al. 2004) . This construct contains three oxytocin receptor estrogen responsive elements located upstream of a luciferase reporter (Adan, Cox et al. 1992) . Monoclonal anti-E-cadherin (32A8; 1:1000 dilution) (#5296) were from Cell Signaling. Monoclonal anti-gamma-tubulin (clone GTU-88; 1:7500 dilution) (#T6557) and 17β-estradiol (E2) were from Sigma-Aldrich. Human PRL was purchased from the National Hormone and Peptide Program (Dr. Parlow, National Institute of Diabetes and Digestive and Kidney Disease, Bethesda, MD, USA).
